# Gene losses and partial deletion of small single-copy regions of the chloroplast genomes of two hemiparasitic *Tax*i*llus* species

**DOI:** 10.1038/s41598-017-13401-4

**Published:** 2017-10-12

**Authors:** Ying Li, Jian-guo Zhou, Xin-lian Chen, Ying-xian Cui, Zhi-chao Xu, Yong-hua Li, Jing-yuan Song, Bao-zhong Duan, Hui Yao

**Affiliations:** 1 The Key Laboratory of Bioactive Substances and Resources Utilization of Chinese Herbal Medicine, Ministry of Education, Institute of Medicinal Plant Development, Chinese Academy of Medical Sciences & Peking Union Medical College, Beijing, 100193 China; 2 Department of Pharmacy, Guangxi Traditional Chinese Medicine University, Nanning, 530200 Guangxi China; 3 College of Pharmaceutical Science, Dali University, Dali, 671000 Yunnan China

## Abstract

Numerous variations are known to occur in the chloroplast genomes of parasitic plants. We determined the complete chloroplast genome sequences of two hemiparasitic species, *Tax*i*llus ch*i*nens*i*s* and *T*. *sutchuenensis*, using Illumina and PacBio sequencing technologies. These species are the first members of the family Loranthaceae to be sequenced. The complete chloroplast genomes of *T*. *chinensis* and *T*. *sutchuenensis* comprise circular 121,363 and 122,562 bp-long molecules with quadripartite structures, respectively. Compared with the chloroplast genomes of *N*i*cot*i*ana tabacum* and *Osyr*i*s alba*, all *ndh* genes as well as three ribosomal protein genes, seven tRNA genes, four *ycf* genes, and the i*nfA* gene of these two species have been lost. The results of the maximum likelihood and neighbor-joining phylogenetic trees strongly support the theory that Loranthaceae and Viscaceae are monophyletic clades. This research reveals the effect of a parasitic lifestyle on the chloroplast structure and genome content of *T*. *chinensis* and *T*. *sutchuenensis*, and enhances our understanding of the discrepancies in terms of assembly results between Illumina and PacBio.

## Introduction

The chloroplast is a key plant cell organelle that carries out photosynthesis^1^. The chloroplast genome is highly conserved and has multiple copies, which means that target genes are expressed at high levels^2,3^. In recent years, the chloroplast genome has increasingly been used as a source of molecular markers^4,5^ and barcoding identification^6,7^, and genomic information from this organelle has been utilized in studies of plant evolution, phylogenetics, and diversity^8,9^. With the rapid development of sequencing and bioinformation technology, an increasing number of plant chloroplast genomes, including medicinal plants, have been determined, such as *Glyc*i*ne max* ^10^, *Soughum b*i*color* ^11^, *Magnol*i*a off*i*c*i*nal*i*s* ^12^, *Taxus ch*i*nens*i*s* var. *ma*i*re*i^13^ and *Astragalus membranaceus* ^14^.

Given that parasitic plants have either lost some or all their photosynthetic capacity, they absorb organic and inorganic nutrients as well as water from their hosts by maintaining a much higher transpiration rate and using specialized parasitic organs called haustoria^15^. Despite their large known diversity, only a few chloroplast genomes from parasitic plants have been obtained. The first complete parasitic plant chloroplast genome to be sequenced was from *Ep*i*fagus v*i*rg*i*n*i*ana* ^16^. All photosynthesis and energy producing genes in this species have been lost, although a few fragments remain as pseudogenes, and the entire chloroplast genome no longer performs photosynthesis^17^. Subsequently sequenced chloroplast genomes include four species from the holoparasitic genus *Cuscuta*, including *C*. *reflexa*, *C*. *gronov*ii, *C*. *exaltata* and *C*. *obtus*i*flora* ^18,19^. Previous studies showed that the chloroplast genome of *Raffles*i*a lagascae* is completely lost^20^. The complete chloroplast genomes of several species within the parasitic family Orobanchaceae have been sequenced and analyzed in recent years, including the completely non-photosynthetic plants, *C*i*stanche desert*i*cola* ^1^, *Phel*i*panche ramosa* ^21^, *Orobanche austroh*i*span*i*ca* ^22^, and *Lathraea squamar*i*a* ^23^. More recently, Petersen *et al*. sequenced and analyzed the complete chloroplast genome of one species of the genus *Osyr*i*s* and three species of the genus *V*i*scum* ^24^. A number of photosynthetic and photorespiratory genes, some protein-coding genes, ribosomal protein genes, transfer RNA (tRNA) genes from some parasitic plants have either been completely lost or pseudogenized^23–25^. Horizontal gene transfer also occurs between donor and recipient in some parasitic plants^1,26^.

Plants within Loranthaceae comprise hemiparasitic species that have retained photosynthesis and have seeds which are widely propagates by birds^27^. The taxonomy of plants within Loranthaceae is controversial, particularly regarding the branching point between these taxa and Viscaceae. To date, the plants in China classified within Loranthaceae have been studied and the results demonstrated that, apart from the hemiparasitic characteristics, significant differences exist in pollen morphology^28^, chemical composition^29^, and DNA molecules^30^, which nevertheless support the theory that Loranthaceae and Viscaceae are branched independently. However, one medicinal plant within Viscaceae, namely, *V*i*scum coloratum* (Kom.) Nakai, is assigned to Loranthaceae in the Chinese Pharmacopoeia^31^.

Approximately 70 genera comprising more than 900 species are classified within Loranthaceae^32^. Most of these plant species primarily live in tropical and subtropical regions, with 8 genera and 51 species (18 endemic) found in China^33^. Of these genera, the hemiparasitic plant genus *Tax*i*llus* consists of species with degenerated chloroplasts and restricted photosynthetic capacity. Specifically, *T*. *chinensis* is used in traditional Chinese herbal medicine and is recorded in the Chinese Pharmacopoeia^31^. Another species, namely, *T*. *sutchuenensis*, is used in folk medicine. These two medicinal plants are commonly used to treat diseases, such as rheumatism, hypertension, and fetal irritability^34,35^. The recorded hosts of *T*. *chinensis* and *T*. *sutchuenensis* include species within Moraceae, Rutaceae, Aceraceae, Anacardiaceae, Euphorbiaceae, Rosaceae, Theaceae and rarely Taxodiaceae^33^.

The third-generation sequencing platform PacBio is based on single-molecule real-time (SMRT) sequencing technology. The main advantage of this sequencing approach is the long read length, generating read lengths of over 10 kb on average, with some reads possibly reaching up to 60 kb^36–38^. Previous studies have demonstrated that the long read lengths provide many benefits in genome assembly, including generating longer contigs and fewer unresolved gaps^39^. PacBio has been successfully applied in a number of chloroplast genome sequencing projects involving three species of *Fr*i*t*i*llar*i*a* ^38^, *Acon*i*tum barbatum* var. *puberulum* ^40^, and *Swert*i*a mussot*ii^41^. However, PacBio has high rates of random error in single-pass reads^37^. In this study, the chloroplast genome sequence of *T*. *chinensis* was sequenced using second-generation Illumina platform and third-generation PacBio system to verify the accuracy of the genome sequence.

We report the complete chloroplast genome of *T*. *chinensis* and *T*. *sutchuenensis*, which are the first two sequences completed within Loranthaceae. We also present a comparative analysis of the genetic changes together with chloroplast genomes of five other species, including the previously reported sequence of *V*i*scum m*i*n*i*mum*, to determine the effect of a parasitic lifestyle on chloroplast structure and the genome content. We also analyzed the phylogenetic relationships of *T*. *chinensis* and *T*. *sutchuenensis* within Dicotyledoneae based on the complete chloroplast genomes to provide baseline data for systematic classification of Loranthaceae.

## Results

### Chloroplast Genome Structures of *T*. *chinensis* and *T*. *sutchuenensis*

**Figure 1:**
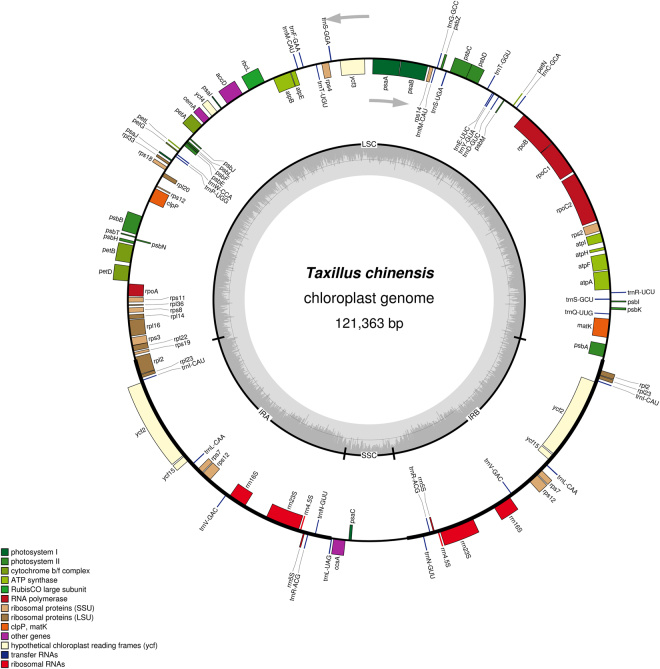
Gene map of the complete chloroplast genome of *T*. *chinensis*. Genes on the inside of the circle are transcribed clockwise, while those outside are transcribed counter clockwise. The darker gray in the inner circle corresponds to GC content, whereas the lighter gray corresponds to AT content.

**Figure 2:**
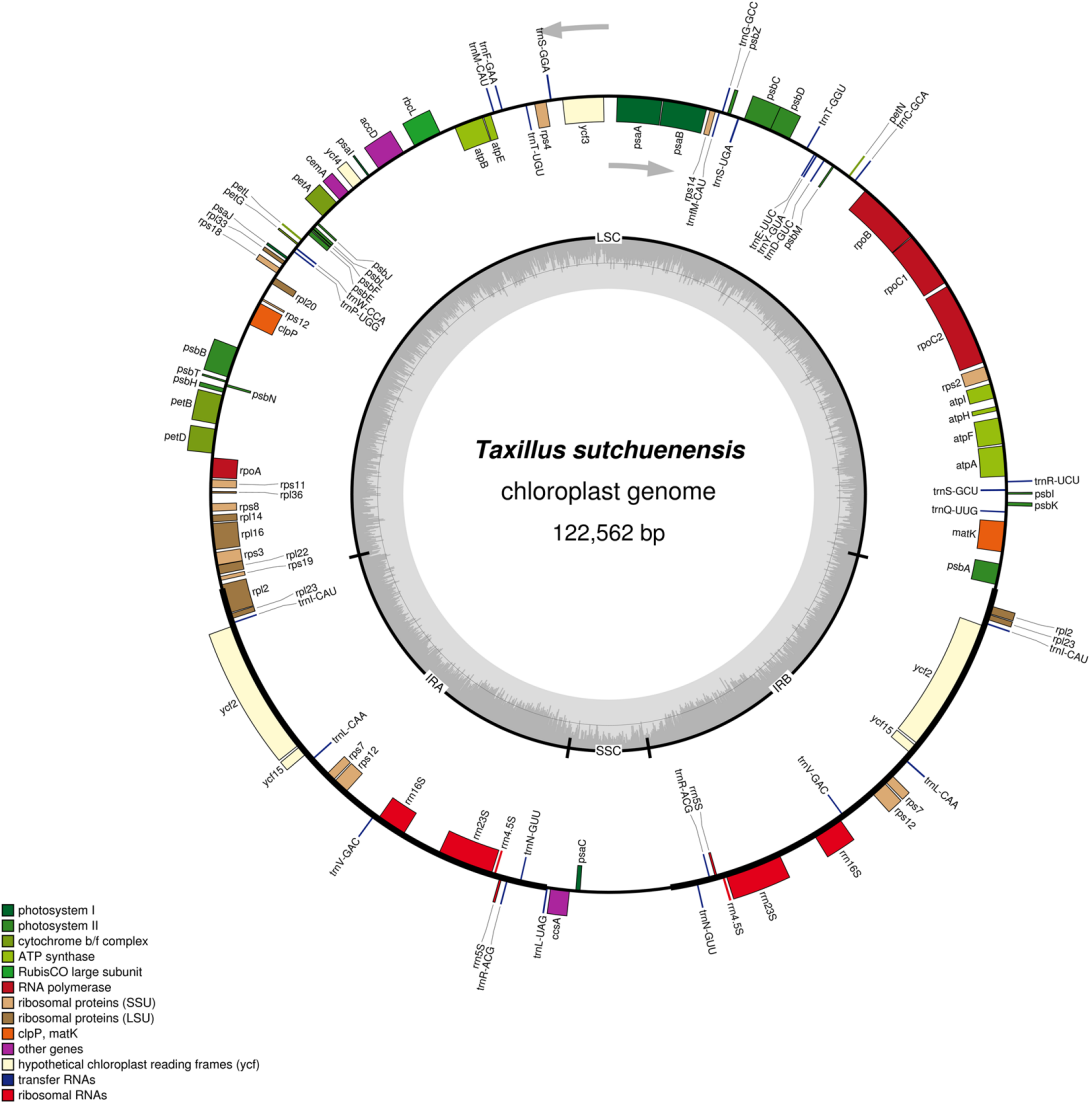
Gene map of the complete chloroplast genome of *T*. *sutchuenensis*. Genes on the inside of the circle are transcribed clockwise, while those outside are transcribed counter clockwise. The darker gray in the inner circle corresponds to GC content, whereas the lighter gray corresponds to AT content.

Results show that the chloroplast genome sequence of *T*. *chinensis* is a circular molecule that is 121,363 bp in length, which can be divided into a large single-copy (LSC) region of 70,357 bp and a small single-copy (SSC) region of 6,082 bp, and separated by a pair of inverted repeats (IRa and IRb) each 22,462 bp in length (Fig. 1). This sequence, which was assembled using the reads obtained by the Illumina sequencing platform, is 121,363 bp in length. By contrast, the sequence assembled using the reads obtained by the PacBio system is 12 bp shorter than that assembled from the reads obtained by the Illumina platform. After verification using PCR, we found that the complete chloroplast genome of *T*. *chinensis* is consistent with the assembly results obtained using the reads from second-generation sequencing. The chloroplast genome of *T*. *sutchuenensis* is extremely similar to that of *T*. *chinensis* in size and genomic structure; it is 122,562 bp in length and retains a typical structure comprising a LSC (70,630 bp), a SSC (6,102 bp), and two IRs, each having 22,915 bp (Fig. 2). The complete and correct chloroplast genome sequences of *T*. *chinensis* and *T*. *sutchuenensis* were deposited in GenBank under accession numbers KY996492 and KY996493, respectively.

Data reveal that both species have a GC content of 37.3%, which is unevenly distributed across the whole chloroplast genome. In both cases, the GC content of the IR regions exhibits the highest values across the complete chloroplast genome, 43.0% in *T*. *chinensis* and 42.8% in *T*. *sutchuenensis*, respectively. This high GC content in IR regions is the result of four rRNA genes (*rrn16*, *rrn23*, *rrn4*.5 and *rrn5*) that occur in this region^42^. In addition, after the LSC, which has a GC content of 34.7%, lowest values of 26.2% are seen in SSC regions.

A total of 106 genes were identified in each genome, which include 66 protein-coding genes, 28 tRNAs, 8 rRNAs, and 4 pseudogenes. Simultaneously, we compared these two *Tax*i*llus* species with autotrophic plants, including *N*i*cot*i*ana tabacum* and *Osyr*i*s alba*. Genes encoding subunits of the NAD(P)H dehydrogenase complex (*ndh* genes) were missing from the chloroplast genome of the two species, whereas three genes for ribosomal proteins (*rpl32*, *rps15*, and *rps16*), seven tRNA genes (*trnA-UGC*, *trnG-UCC*, *trnH-GUG*, *trnL-GAU*, *trnK-UUU*, *trnL-UAA*, and *trnV-UAC*), four *ycf* genes (*ycf1*, *ycf5*, *ycf9*, and *ycf10*), and initiation factor gene (i*nfA*) were also lost (Table S1). Two ribosomal protein genes (*rpl16* and *rpl2*) and the duplicate gene *ycf15* have also been pseudogenized because their gene-coding regions are interrupted by deletion, insertions or internal stop codons, while the pseudogene *rpl2* is located in IRb region. We designed the primers to perform PCR to verify the accuracy of the pseudogenes *rpl16*, *ycf15* and *rpl2*. The primer sequences are listed in Supplementary Table S2.

The basic information and gene contents of the chloroplast genomes of *T*. *chinensis* and *T*. *sutchuenensis* compared to other five species are presented in Table 1 and Supplementary Table S1.

**Table 1:** Comparisons among the chloroplast genome characteristics of *T*. *chinensis*, *T*. *sutchuenensis*, and other five species.

**Species**	***Tax*** **i** ***llus ch*** **i** ***nens*** **i** ***s***	***Tax*** **i** ***llus sutchuenens*** **i** ***s***	***V*** **i** ***scum m*** **i** ***n*** **i** ***mum***	***Osyr*** **i** ***s alba***	***Schoepf*** **i** ***a jasm*** **i** ***nodora***	***Ep*** **i** ***fagus v*** **i** ***rg*** **i** ***n*** **i** ***ana***	***N*** **i** ***cot*** **i** ***ana tabacum***
Family	Loranthaceae	Loranthaceae	Viscaceae	Santalaceae	Olacaceae	Orobanchaceae	Solanaceae
Accession No.	KY996492	KY996493	KJ512176	KT070882	KX775962	M81884	Z00044
Genome size(bp)	121,363	122,562	131,016	147,253	118,743	70,028	155,844
LSC length(bp)	70,357	70,630	75,814	84,601	84,168	19,799	86,684
SSC length(bp)	6,082	6,102	9,014	13,972	9,763	4,759	18,482
IR length(bp)	22,462	22,915	23,094	24,340	12,406	22,735	25,339
GC content(%)	37.3	37.3	36.2	37.7	38.1	37.5	37.8
Number of genes	106	106	104	114	112	53	151
Number of protein-coding genes	66	66	66	67	69	10	112
Number of tRNAs	28	28	29	30	35	17	30
Number of rRNAs	8	8	8	8	8	8	8
Number of pseudogenes	4	4	1	9	5	18	1

Introns play an important role in the regulation of gene expression. Introns enhance exogenous gene expression at specific sites within plants at particular times, resulting in desirable agronomic traits^43^. Introns within these two species are similar to other angiosperms^1,44,45^. Results reveal the presence of nine genes containing introns in each chloroplast genome, including *atpF*, *rpoC1*, *ycf3*, *rps12*, *rpl2*, *ψrpl16*, *clpP*, *petB*, and *petD*. In addition, the *ycf3* gene and *rps12* gene each contain two introns and three exons. The *ycf3* gene is located within the LSC, as seen in *Metasequo*i*a glyptostrobo*i*des* ^45^, *Aqu*i*lar*i*a s*i*nens*i*s* ^46^, while the *rps12* gene is specialized for trans-splicing. The 5′ exon is located in the LSC, and the 3′ exon is located in the IR, as is the case in *Panax g*i*nseng* ^44^, *C*. *desert*i*cola* ^1^, and *L*. *squamar*i*a* ^23^. Relevant lengths of exons and introns are listed in Table 2.

**Table 2:** Genes with introns in the chloroplast genomes of *T*. *chinensis* and *T*. *sutchuenensis* as well as the lengths of the exons and introns.

**Species**	**Gene**	**Location**	**Exon1(bp)**	**Intron1(bp)**	**Exon2(bp)**	**Intron2(bp)**	**Exon3(bp)**
*T*. *chinensis*	*atpF*	LSC	150	779	375		
	*clpP*	LSC	335	621	229		
	*petB*	LSC	6	755	642		
	*petD*	LSC	9	718	483		
	*rpl2*	LSC; IR	394	645	437		
	*ψrpl16*	LSC	10	924	397		
	*rps12*	LSC, IR	114	—	232	543	26
	*rpoC1*	LSC	456	752	1617		
	*ycf3*	LSC	127	730	230	771	153
*T*. *sutchuenensis*	*atpF*	LSC	163	753	410		
	*clpP*	LSC	332	634	229		
	*petB*	LSC	6	799	642		
	*petD*	LSC	6	715	483		
	*rpl2*	LSC; IR	399	712	369		
	*ψrpl16*	LSC	9	924	389		
	*rps12*	LSC, IR	114	—	232	539	26
	*rpoC1*	LSC	450	756	1602		
	*ycf3*	LSC	127	759	230	785	153

### Comparative genome analyses

**Figure 3:**
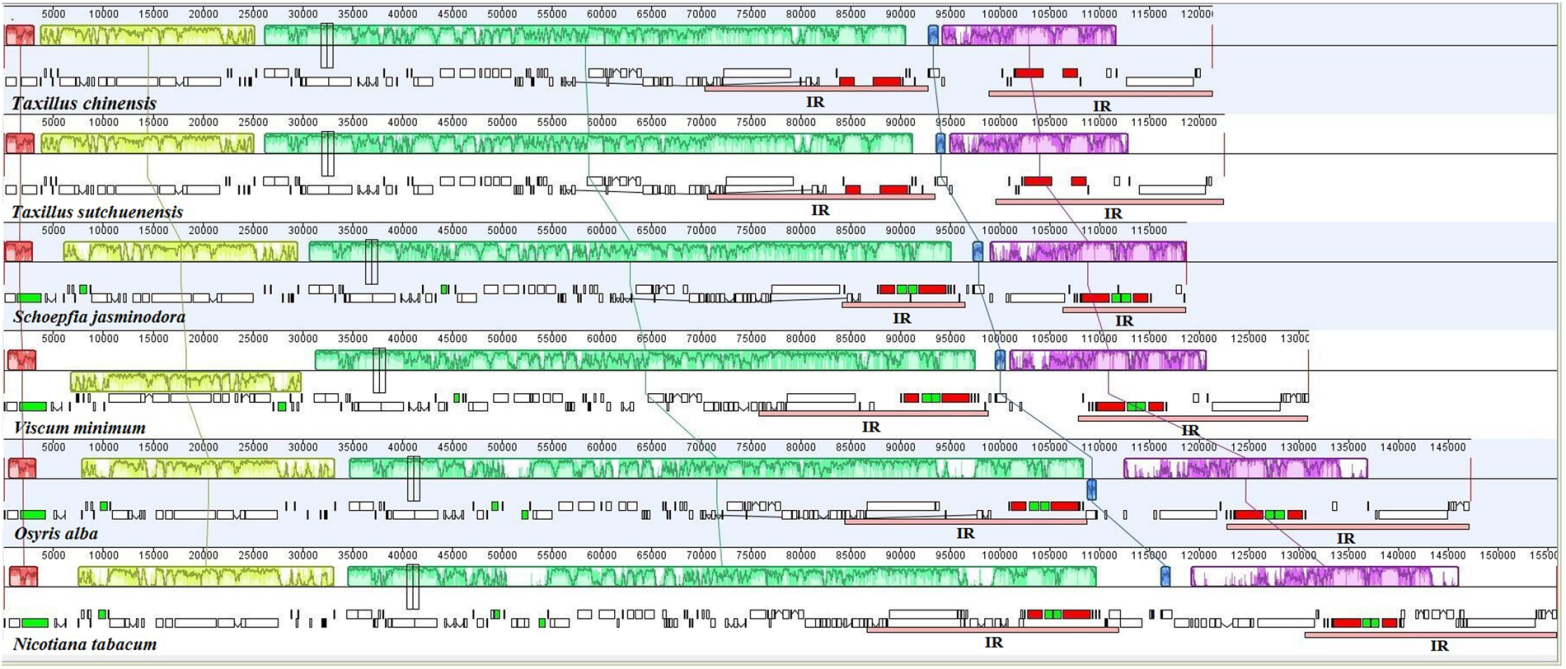
Comparison of the complete chloroplast genomes of six species using the MAUVE algorithm. Local collinear blocks are colored in this figure to indicate syntenic regions, while histograms within each block represent the degree of sequence similarity.

Data plotted using mVISTA (Fig. S1) reveal that non-coding regions of the chloroplast genomes of the two *Tax*i*llus* species are more divergent than their coding counterparts. Moreover, the two IR regions have lower sequence divergence than the LSC and SSC regions. Similar results were obtained in previous research on the complete chloroplast genomes of five Lamiales species^42^ as well as in a comparative study of five *Ep*i*med*i*um* chloroplast genomes^47^. In the present study, *rpl16* gene is the most divergent of the coding regions, probably because of pseudogenization. Thus, we conducted a series of linear rearrangement comparisons across the complete chloroplast genome sequences of six species (*T*. *chinensis*, *T*. *sutchuenensis*, *S*. *jasm*i*nodora*, *V*. *m*i*n*i*mum*, *O*. *alba* and *N*. *tabacum*) aligned in Geneious using the Mauve algorithm (Fig. 3). The comparisons reveal the presence of two structural variants, including an approximately 24-kb-long inversion within the LSC region of the *V*. *m*i*n*i*mum* chloroplast genome and an approximately 3-kb-long inversion in the SSC region of the *O*. *alba* chloroplast genome, which is consistent with a previous report^24^. The lengths of the IR regions in our two species are also similar to that of other plants, with the exception of *S*. *jasm*i*nodora* ^48^ where they are much shorter (at least 10 kb) than the length of the IR regions of the five species considered here, including *T*. *chinensis* and *T*. *sutchuenensis*.

### Codon Usage

**Figure 4:**
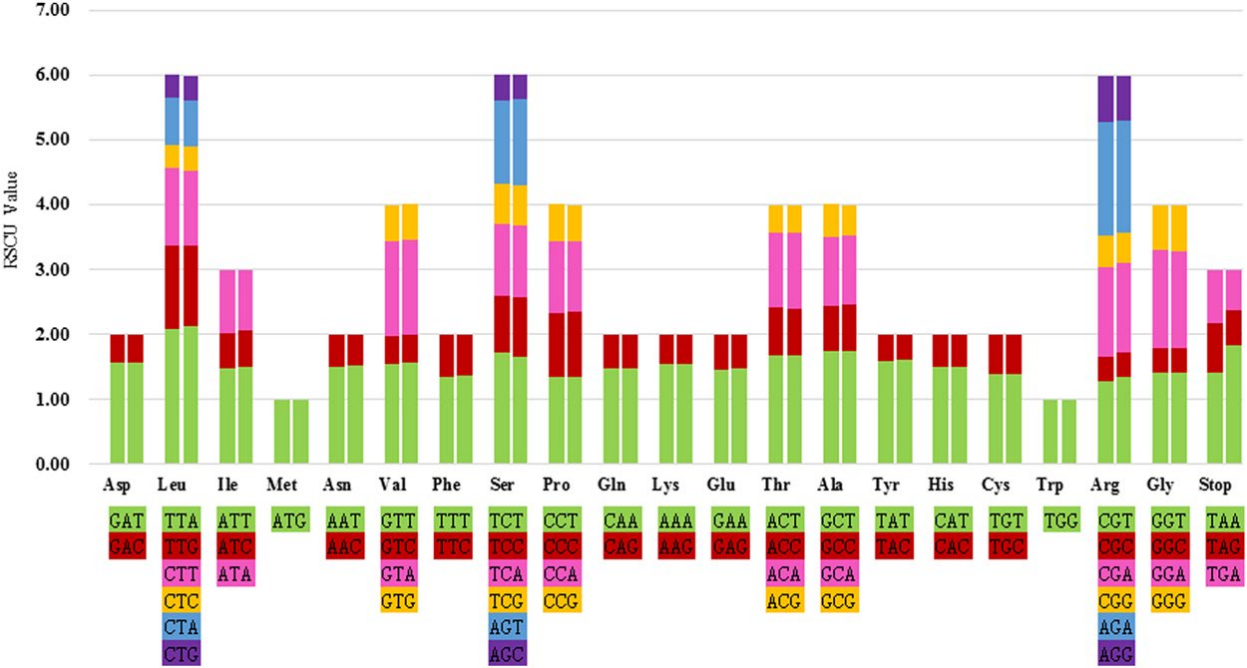
Codon content of 20 amino acid and stop codons in all protein-coding genes of the chloroplast genomes of two species. The histogram on the left-hand side of each amino acid shows codon usage within the *T*. *chinensis* chloroplast genome, while the right-hand side illustrates the genome of *T*. *sutchuenensis*.

The calculations for the codon usage of protein-coding genes within *T*. *chinensis* and *T*. *sutchuenensis* chloroplast genomes are summarized in Fig. 4 and Supplementary Table S3. Results reveal the presence of 63 codons encoding 20 amino acids within the chloroplast protein-coding genes of these two species; of these, 1711 encode leucine and 191 encode cysteine, which are respectively the most and least prevalent amino acids in *T*. *chinensis* chloroplast genome. Results also reveal that most of the amino acid codons have preferences, with the exception of methionine and tryptophan. Moreover, usage is generally biased toward A or T with high relative synonymous codon usage (RSCU) values, including TTA (2.12) in leucine, TAT (1.62) in tyrosine, and the stop-codon TAA (1.84) in the *T*. *sutchuenensis* chloroplast genome (Supplementary Table S3). The data presented in Fig. 4 illustrates that the RSCU value increases with the quantity of codons that code for a specific amino acid. High codon preference, especially a strong AT bias in codon usage, is very common in other land plant chloroplast genomes^42,44^. The present results are similar to the chloroplast genomes of *A*. *s*i*nens*i*s* ^46^ and species within the genus *Ulmus* ^49^ in terms of codon usage.

### Simple Sequence Repeats (SSRs) Analyses

SSRs are ubiquitous throughout genomes and are also known as microsatellites. SSRs comprise tandem repeated DNA sequences that consist of between one and six repeat nucleotide units^50^. As such, SSRs are widely used as molecular markers in species identification, population genetics, and phylogenetic investigations because they exhibit high levels of polymorphism^51–53^. In total, 195 and 198 SSRs are identified within the chloroplast genomes of *T*. *chinensis* and *T*. *sutchuenensis*, respectively (Table 3; Supplementary Tables S4–S5), which mainly comprise mononucleotide repeats encountered 146 and 139 times in each case. In addition, A/T mononucleotide repeats (93.9% and 96.4%, respectively; Table 3) are the most common, while the majority of dinucleotide repeat sequences comprise AT/TA repeats (59.5% and 67.3%, respectively; Table 3). Results show that SSRs within the chloroplast genomes of *T*. *chinensis* and *T*. *sutchuenensis* are dominated by AT-rich repetitive motifs, which is consistent with the fact that AT content is also very high (62.7%) in these species. This result is also in agreement with previous studies showing higher proportions of polyadenine (polyA) and polythymine (polyT) relative to polycytosine (polyC) and polyguanine (polyG) within the chloroplast SSRs in many plants^6^.

**Table 3:** Types and amounts of SSRs in the *T*. *chinensis* and *T*. *sutchuenensis* chloroplast genomes.

**SSR type**	**Repeat unit**	**Amount**		**Ratio(%)**	
		*T*. *chinensis*	*T*. *sutchuenensis*	*T*. *chinensis*	*T*. *sutchuenensis*
mono	A/T	138	134	93.9	96.4
	C/G	9	5	6.1	3.6
di	AC/GT	3	4	7.2	7.7
	AG/CT	14	13	33.3	25
	AT/TA	25	35	59.5	67.3
tri	AAG/CTT	2	2	100	50
	AAT/ATT	0	2	0	50
tetra	AAAC/GTTT	1	0	25	0
	AAAG/CTTT	1	0	25	0
	AATC/ATTG	1	0	25	0
	ACAG/CTGT	1	1	25	50
	AAGT/ACTT	0	1	0	50
penta	AATAT/ATATT	1	1	100	100

### Phylogenetic Analyses

**Figure 5:**
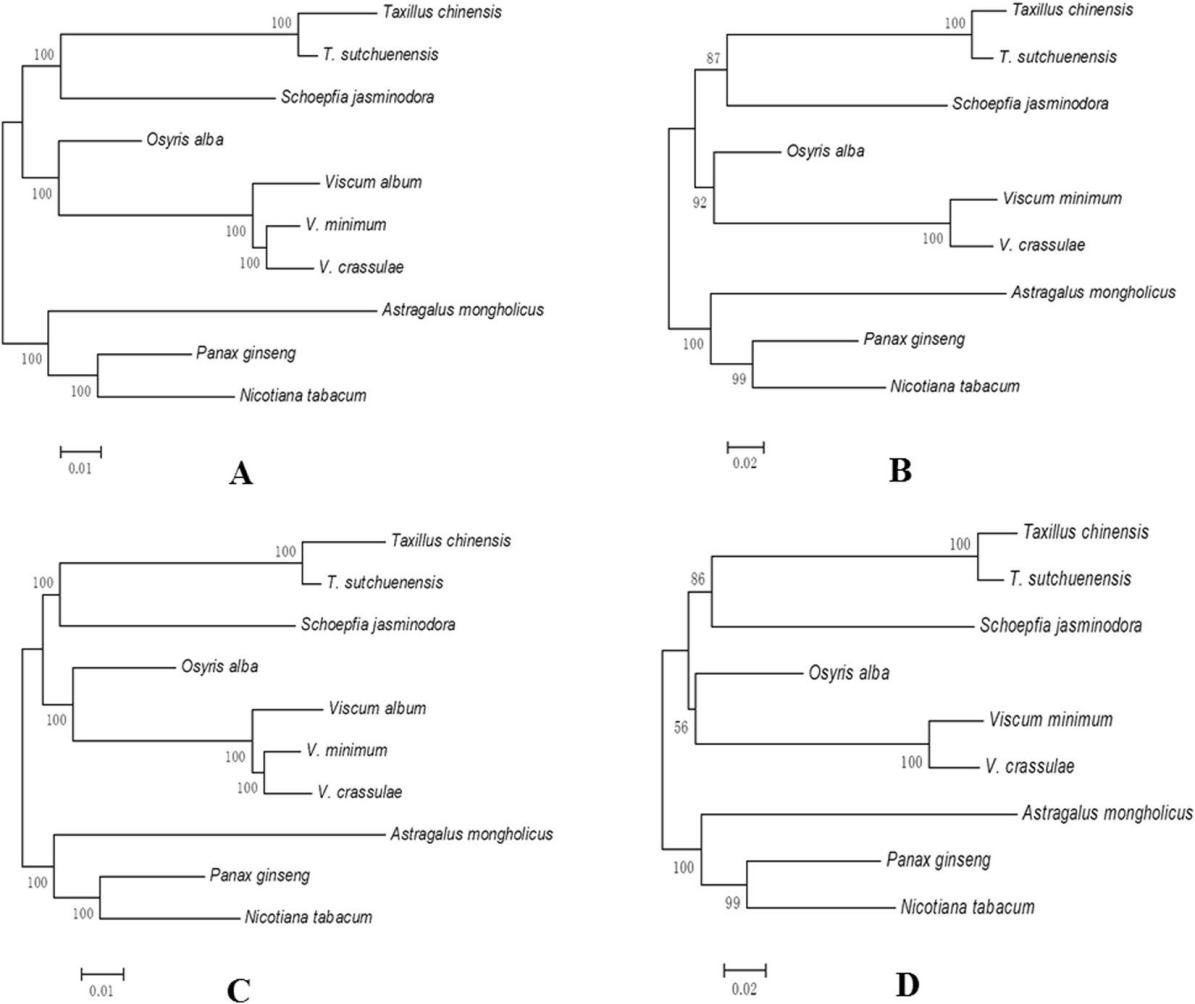
Phylogenetic trees constructed using two methods based on two datasets from different species. (**A**) ML tree based on 54 protein-coding genes; (**B**) ML tree based on *matK* genes; (**C**) NJ tree based on 54 protein-coding genes; (**D**) NJ tree based on *matK* genes. Number at nodes are values for bootstrap support.

Phylogenetic trees were constructed using two methods based on two datasets from different species (Fig. 5). Results revealed extremely similar tree topologies from each dataset irrespective of the method used, as supported by high bootstrap values. All nodes in our maximum likelihood (ML) and neighbor-joining (NJ) trees based on 54 protein-coding genes have 100% bootstrap support values, whereas four out of six nodes that received bootstrap values of ≥99% were recovered in both sets of trees when *matK* genes were used for analyses. All nodes in all phylogenetic trees received higher than 50% bootstrap support. All four phylogenetic trees showed that *T*. *chinensis* and *T*. *sutchuenensis* are sister taxa with respect to *S*. *jasm*i*nodora* (Olacaceae), whereas the three species within genus *V*i*scum* group with *Osyr*i*s alba* (Santalaceae) and all Santalales species are clustered within a lineage distinct from the outgroup.

## Discussion

Numerous variations occur in the chloroplast genomes of parasitic plants. To date, however, most investigations on these genomes in parasitic and heterotrophic plants focused on nonphotosynthetic species^24^. For instance, some complete chloroplast genomes of holoparasitic plants from Orobanchaceae were reported^1,21,22^. A small number of hemiparasitic plants within Santalales and other groups have been studied^24,48^. In this study, the complete chloroplast genomes of *T*. *chinensis* and *T*. *sutchuenensis* from Santalales were assembled, annotated, and analyzed.

Gene loss events have occurred within the chloroplast genomes of most parasitic plants and in a handful of autotrophic species^21,54^. Previous work has shown that genes of *chlB*, *chlL*, *chlN*, and *trnP-GGG* have been lost from the chloroplast genomes of most flowering plants^55^, whereas gene i*nfA*, which codes for a translation initiation factor, is either missing or has been transferred in many plants^56^, including the two species observed in this study. All *ndh* genes have been lost from the chloroplast genomes of *T*. *chinensis* and *T*. *sutchuenensis*, similar to the case of *Cuscuta gronov*ii and *C*. *obtus*i*flora* ^18,19^. Similarly, nine out of eleven *ndh* genes have been pseudogenized within the chloroplast genome of *L*. *squamar*i*a* ^23^. This degree of *ndh* gene degradation is not only observed in heterotrophic organisms, but also in many autotrophic plants, including Orchidaceae^57,58^, Geraniaceae^59^ and Cactaceae^60^. Kim *et al*. reported that losses of *ndh* genes in angiosperms are usually associated with nutritional status and/or extensive rearrangements of chloroplast structures^57^. Also, *ndh* genes loss events and pseudogenization have occurred in reported chloroplast genomes of parasitic plants, regardless of the degree of degradation in photosynthetic capacity^1,23,24,48^. As a result, studies suggested that *ndh* genes were first lost in the transformation from autotrophy to heterotrophy^18,61^.

In this study, seven transfer RNA genes, including *trnK-UUU*, have been lost from the chloroplast genomes of both species. Although similar tRNA losses have commonly occurred in most plants (Supplementary Table S1), the *trnK-UUU* gene, which is generally absent from most parasitic plants, is completely preserved (including its intron *matK* gene) within the chloroplast genome of *C*i*stanche desert*i*cola* ^1^. Li *et al*. suggested that tRNA genes from the chloroplast genome were lost later than photosynthesis genes^1^.

A pseudogene, which is a defective copy of the functional gene, is widespread in the chloroplast genome of plants and has lost the normal protein coding function^1,18,19,62^. Loss of genic normal activity is generally caused by mutations inhibiting gene expression. Pseudogenes not only demonstrate gene mutation accumulation but are also associated with gene expression and regulation^63^. Four pseudogenes exist in the chloroplast genomes of *T*. *chinensis* and *T*. *sutchuenensis*; these pseudogenes include *rpl16*, *rpl2* and *ycf15* (duplicate gene). The gene of *ycf15* has been pseudogenized in many plants, including *S*. *jasm*i*nodora* ^48^, *C*. *reflexa* ^19^ and *C*. *exaltata* ^18^. Genes of *rpl16* and *rpl2* exist in most plants as functional genes, whereas they have been pseudogenized in the current study.

A previous study pointed out that one early response of the chloroplast genomes to the evolution of a parasitic lifestyle was condensation via losses in numerous non-coding and unimportant regions; this event resulted in reduction of chloroplast genome size^19^. Although gene loss can be regarded as a terminal evolutionary step, accumulation of point mutations leading to pseudogenization nevertheless occurred at previous steps^24^.

Second-generation sequencing technology provides an efficient, novel, and rapid method for whole-genome sequencing^12,64,65^. SMRT sequencing, which is combined with circular consensus sequencing (CCS), provides multiple reads of individual templates^40^. Wu *et al*.^66^ have compared three generations of sequencing technologies (Sanger, Illumina and PacBio) on chloroplast genome assembly. Results demonstrated that long reads from PacBio showed potential for highly accurate “finished” genomes. However, the accuracy between second-generation and third-generation sequencing platforms was not compared thoroughly. In the present study, the complete chloroplast genome sequence of *T*. *chinensis* was sequenced using Illumina and PacBio platforms. Discrepancies in terms of assembly results between Illumina and PacBio were detected using PCR-based conventional Sanger sequencing, and the quality is very high. Results revealed that in PacBio platform, the error rate is high in homopolymers when the number of repeat units of a mononucleotide is higher than or equal to six. In the chloroplast genome of *T*. *chinensis*, polyA/T and polyC/G (repeat higher than or equal to six) included 509 and 72 sites, respectively. Although A/T mononucleotide repeats are the most common types (Table 3), these errors are mainly present in structures of polyC and polyG (Table 4). Among the 14 errors, 12 were G/C deletions, 1 was A/T deletion, and 1 was A/T insertion. All errors differed in terms of only one base.

**Table 4:** Discrepancies in assembly results obtained using Illumina and PacBio.

**No**.	**Sites (bp)**	**Repeat unit**	**Number of repeat unit**		**Location**
			**Illumina**	**PacBio**	
1	4615	C	10	9	intergenic region
2	17326	C	9	8	introns
3	17952	C	7	6	introns
4	27768	G	7	6	*psbC*
5	32700	C	6	5	*psbA*
6	41040	C	10	9	intergenic region
7	45020	G	8	7	*rbcL*
8	51046	T	6	7	intergenic region
9	55514	C	10	9	intergenic region
10	58056	A	9	8	intergenic region
11	87056	G	7	6	intergenic region
12	90138	C	8	7	intergenic region
13	101590	G	8	7	intergenic region
14	104671	C	7	6	intergenic region

As a result of multiple comparisons (Table 1 and Fig. 3), we observed that complete lengths of the chloroplast genomes of *T*. *chinensis* and *T*. *sutchuenensis* are similar to those of *S*. *jasm*i*nodora* and *V*. *m*i*n*i*mum*, whereas the lengths of SSC regions are much smaller (at least 3 kb). These regions, which contain most *ndh* genes, also encapsulate the largest variation within the chloroplast genome^67^ and have undergone dramatic reductions in some parasitic plants, including *L*. *clandest*i*ne* ^68^. Previous studies have demonstrated that positions of IR junction and SSC region are correlated with degeneration of *ndhF* and *ycf1* genes^57,69^. Loss of *ycf1* and all *ndh* genes (including *ndhF*), as revealed by this study may explain why SSC chloroplast genome regions of the two considered species are shorter than those of others.

Chloroplast genomes have provided significant data for evolutionary, taxonomic, and phylogenetic studies^46^. Specifically, the chloroplast gene of *matK* has been widely utilized in plant phylogenetic analyses^70,71^. In this study, we constructed phylogenetic trees using ML and NJ methods based on *matK* and 54 protein-coding genes commonly present in the chloroplast genomes of ten species, including two medicinal hemiparasites in the current study. Phylogenetic results are extremely consistent, irrespective of method and dataset. All phylogenetic results strongly support the theory that Loranthaceae and Viscaceae diverged independently from one another. Phylogenetic results discussed in the present study are broadly consistent with those of a previous research, which utilized chloroplast *trnL* intron sequences to investigate inter-familial relationships within Santalales^30^.

## Conclusions

The complete chloroplast genome sequences of traditional medicinal hemiparasites *T*. *chinensis* and *T*. *sutchuenensis* were obtained and analyzed. Results of this study revealed effects of parasitic lifestyle on chloroplast structure and genome content in these species and enhanced understanding of phylogenetic positions and relationships of *T*. *chinensis* and *T*. *sutchuenensis*. This research also showed that sequences assembled using reads obtained by the Illumina platform is more accurate than those from PacBio.

## Materials and Methods

### Plant Material, DNA Extraction, and Sequencing

Fresh leaves of *T*. *chinensis* and *T*. *sutchuenensis* were collected from Qinzhou City in Guangxi Province and from Lichuan City in Hubei Province, respectively. All samples were identified by Professor Yulin Lin, who is based at the Institute of Medicinal Plant Development (IMPLAD), Chinese Academy of Medical Sciences & Peking Union Medical College. The voucher specimens were deposited in the herbarium of IMPLAD. Approximately 100 g of samples frozen in −80 °C were used to extract total genomic DNA using DNeasy Plant Mini Kit (Qiagen Co., Germany). DNA quality was assessed based on electrophoresis and optical density results. DNA of two species was used to generate libraries with average insert size of 500 bp and sequenced using Illumina Hiseq X in accordance with standard protocol. Approximately 4.4 Gb of raw data from *T*. *chinensis* and 3.7 Gb from *T*. *sutchuenensis* using Illumina sequencing platform were generated with 150 bp paired-end read lengths. To compare PacBio with Illumina sequencing technology when employed in chloroplast genome study, we sequenced a PacBio shotgun library of *T*. *chinensis* with an insert size of 3 kb on PacBio RS II platform using P6-C4 chemistry (Pacific Biosciences, Menlo Park, CA, USA). A total of 24,590 CCS reads with a length of 67,512,059 bp were obtained from one SMRT cell, and these reads were used for assembly. Assembly results showed that 13.6% of chloroplast sequences were detected in total data, revealing percentage of chloroplast DNA in total DNA during DNA extraction experiment.

### Chloroplast Genome Assembly

Low-quality reads resulting from all samples were trimmed using the software Trimmomatic^72^. The trimmed reads included a mixture of data from nuclear and organelle genomes. We used the chloroplast genome sequence of *V*i*scum m*i*n*i*mum*, which was downloaded from GenBank to establish a Basic Local Alignment Search Tool (BLASTn) database. Then all trimmed reads were mapped onto this database, and the mapped reads were extracted from raw data based on coverage and similarity. Extracted reads were assembled to contigs using SOAPdenovo2^73^. SSPACE^74^ was used to construct the scaffold of the chloroplast genome, and GapCloser^73^ was used to fill gaps. Reads sequenced using PacBio system were used to assemble the chloroplast genome according to the strategy described by Xiang *et al*.^41^. Assembly results obtained using Illumina and PacBio (Table 4) differed in terms of 14 sites, which are all homopolymers and mainly located at intergenic regions. To detect these discrepancies, we performed PCR-based conventional Sanger sequencing. The primer sequences are listed in Supplementary Table S6.

### Genome Annotation and Structural Analyses

To verify accuracy, including boundaries of single copy and IR regions of assembled sequences, we designed a series of PCR primers (Supplementary Table S7). Annotations of genome sequences of two *Tax*i*llus* species were performed using the online software Dual Organellar GenoMe Annotator (DOGMA, http://dogma.ccbb.utexas.edu/)^75^ and CPGAVAS^76^ with default settings and checked manually. We then used the software tRNAscan-SE^77^ to annotate tRNA genes. Boundaries of genes, introns/exons and coding regions were verified using BLAST versus reference sequences. Circular chloroplast genome map was constructed using an online program Organellar Genome DRAW (OGDRAW) v1.2^78^, and subsequently modified manually. GC content was analyzed using the software MEGA 6.0^79^. Genome comparisons between *T*. *chinensis* and *T*. *sutchuenensis* were performed and plotted using the mVISTA program^80^. The whole-genome alignment for chloroplast genomes of six species, including *T*. *chinensis*, *T*. *sutchuenensis*, *S*. *jasm*i*nodora*, *V*. *m*i*n*i*mum*, *O*. *alba* and *N*. *tabacum*, was performed using the algorithm MAUVE V2.3.1^81^ in the software Geneious v10.1.2 (Biomatters Ltd., http://www.geneious.com/).

### Codon Usage and SSRs Analyses

RSCU value, the ratio between frequency of use and expected frequency of a particular codon, is a simple method for detecting non-uniform synonymous codon usage (SCU) within a coding sequence^82^. In the present study, utilizing the RSCU ratio, we performed statistical analyses to investigate the distribution of codon usage with the software CodonW (http://codonw.sourceforge.net/), applying a 1.00 value for no preference. In addition, a value less than 1.00 refers to a frequency of use that is less than expected, whereas a value higher than 1.00 indicates codons that are more frequently used than expected. Potential SSRs were exploited using the software MISA (http://pgrc.ipk-gatersleben.de/misa/), with parameters set to encompass the number of repeat units of a mononucleotide SSR higher than or equal to eight; followed by higher than or equal to four repeat units for di- and tri-nucleotide SSRs; and higher than or equal to three repeat units for tetra-, penta- and hexa-nucleotides, respectively. In this study, we mainly searched for complete repetitive SSR loci, treating cycled or reverse complementary SSRs as the same type.

### Phylogenetic Analyses

To determine phylogenetic positions of *T*. *chinensis* and *T*. *sutchuenensis* within Santalales, we analyzed the chloroplast genomes of ten species, encompassing five other taxa within this lineage, *V*. *album* (accession number: KT003925), *V*. *crassula* (KT070881), *V*. *m*i*n*i*mum* (KJ512176), *O*. *alba* (KT070882), and *S*. *jasm*i*nodora* (KX775962). We also used the chloroplast genomes of *P*. *g*i*nseng* (AY582139), *N*. *tabacum* (Z00044), and *Astragalus monghol*i*cus* (KU666554) as outgroups, and constructed phylogenetic trees using ML and NJ methods in the software MEGA 6.0^79^ with 1000 bootstrap replicates employing 54 protein-coding genes commonly present in the ten species and *matK* genes. ML analysis was conducted based on the Tamura-Nei model using a heuristic search for initial trees. This most appropriate model was determined by Modeltest 3.7^83^. NJ trees were performed with NJ method^84^, and evolutionary distances were computed using the Kimura 2-parameter method^85^.

## Electronic supplementary material

SI-Gene losses and partial deletion of small single-copy regions of the chloroplast genomes of two hemiparasitic Taxillus species
