# Supplementary material for: Gene losses and partial deletion of small single-copy regions of the chloroplast genomes of two hemiparasitic Taxillus species
Source: Sci Rep. 2017 Oct 12;7:12834. doi: 10.1038/s41598-017-13401-4 (PMC5638910; doi:10.1038/s41598-017-13401-4)
Supplement: Supplementary file 1 — SI-Gene losses and partial deletion of small single-copy regions of the chloroplast genomes of two hemiparasitic Taxillus species [file 41598_2017_13401_MOESM1_ESM.pdf]

Gene losses and partial deletion of small single-copy regions of the chloroplast genomes of two hemiparasitic *Taxillus* species

Ying Li[1+], Jian-guo Zhou[1+], Xin-lian Chen[1], Ying-xian Cui[1], Zhi-chao Xu[1], Yong-hua Li[2], Jing-yuan Song[1], Bao-zhong Duan[3] and Hui Yao[1*]

[1]The Key Laboratory of Bioactive Substances and Resources Utilization of Chinese Herbal Medicine, Ministry of Education, Institute of Medicinal Plant Development, Chinese Academy of Medical Sciences & Peking Union Medical College, Beijing, 100193, China.
[2] Department of Pharmacy, Guangxi Traditional Chinese Medicine University, Nanning, 530200, Guangxi, China.
[3] College of Pharmaceutical Science, Dali University, Dali, 671000, Yunnan, China.
*Correspondence: scauyaoh@sina.com; Tel: +86-10-57833194
[+] These authors contributed equally to this work.

Supplementary Material:

Figure S1: Comparison between the chloroplast genomes of two *Taxillus* species using the software mVISTA;

Table S1: Gene contents within the chloroplast genomes of *T. chinensis* compared to other five species;

Table S2. Primer sequences at the verification of pseudogenes;

Table S3: Codon usage within the chloroplast genomes of *T. chinensis* and *T. sutchuenensis*;

Table S4: Simple sequence repeats (SSRs) within the *T. chinensis* chloroplast genome;

Table S5: Simple sequence repeats (SSRs) within the *T. sutchuenensis* chloroplast genome;

Table S6: Primer sequences at the discrepancies in assembly results obtained using Illumina and PacBio;

Table S7: Primer sequences at the boundaries between single copy and IR regions.

Figure S1. Comparison between the chloroplast genomes of two *Taxillus* species using the software mVISTA.

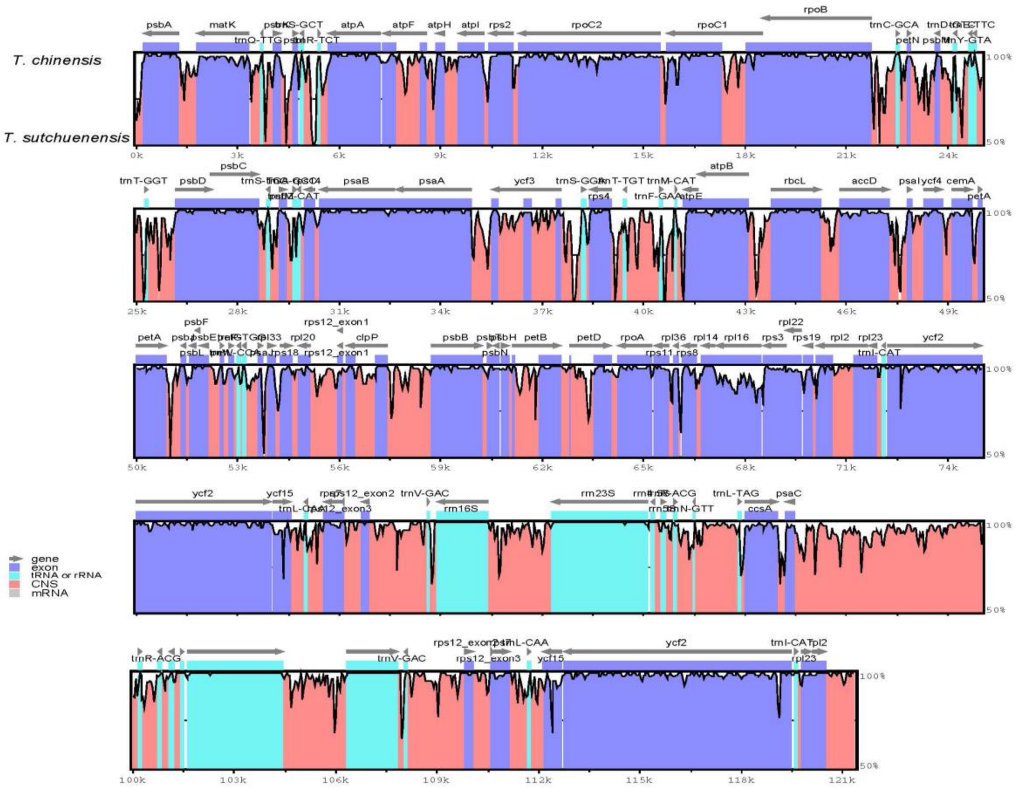

Table S1. Gene contents within the chloroplast genomes of *T. chinensis* compared to other five species.

Genes	*Nicotiana tabacum*	*Osyris alba*	*Schoepfia jasminodora*	*Taxillus chinensis*	*Viscum minimum*	*Epifagus virginiana*
accD	+	+	+	+	+	+
atpA	+	+	+	+	+	Ψ
atpB	+	+	+	+	+	Ψ
atpE	+	+	+	+	+	-
atpF	+	+	+	+	+	-
atpH	+	+	+	+	+	-
atpI	+	+	+	+	+	-
ccsA	-	+	+	+	+	-
cemA	-	+	+	+	+	-
clpP	+	+	+	+	+	+
infA	Ψ	Ψ	+	-	-	+
matK	+	+	+	+	+	+
ndhA	+	Ψ	Ψ	-	-	-
ndhB	+	Ψ	-	-	Ψ	Ψ
ndhC	+	Ψ	-	-	-	-
ndhD	+	Ψ	-	-	-	-
ndhE	+	Ψ	-	-	-	-
ndhF	+	-	-	-	-	-
ndhG	+	Ψ	-	-	-	-
ndhH	+	Ψ	-	-	-	-
ndhI	+	-	-	-	-	-
ndhJ	+	-	-	-	-	-
ndhK	+	Ψ	-	-	-	-
petA	+	+	+	+	+	-
petB	+	+	+	+	+	-
petD	+	+	+	+	+	-
petG	+	+	+	+	+	-
petL	+	+	+	+	+	-
petN	-	+	+	+	+	-
psaA	+	+	+	+	+	-
psaB	+	+	+	+	+	-
psaC	+	+	+	+	+	-
psaI	+	+	+	+	+	-
psaJ	+	+	+	+	+	-
psbA	+	+	+	+	+	Ψ
psbB	+	+	+	+	+	Ψ
psbC	+	+	+	+	+	-
psbD	+	+	+	+	+	-
psbE	+	+	+	+	+	-
psbF	+	+	+	+	+	-
psbH	+	+	+	+	+	-
psbI	+	+	+	+	+	-
psbJ	+	+	+	+	+	-
psbK	+	+	+	+	+	-
psbL	+	+	+	+	+	-
psbM	+	+	+	+	+	-
psbN	+	+	+	+	+	-
psbT	+	+	+	+	+	-
psbZ	-	+	+	+	+	-
rbcL	+	+	+	+	+	Ψ
rp132	+	-	-	-	-	-
rpl12	-	-	-	-	-	+
rpl14	+	+	+	+	+	Ψ
rpl16	+	+	+	Ψ	+	+
rpl2	+	+	+	+	+	+
rpl20	+	+	+	+	+	+
rpl22	+	+	+	+	+	-
rpl23	+	+	+	+	+	Ψ
rpl32	-	+	+	-	+	-

rpl33	+	+	+	+	-	+
rpl36	+	+	+	+	+	+
rpoA	+	+	+	+	+	Ψ
rpoB	+	+	+	+	+	-
rpoC1	+	+	+	+	+	-
rpoC2	+	+	+	+	+	-
rps11	+	+	+	+	+	+
rps12	+	+	+	+	+	+
rps14	+	+	+	+	+	+
rps15	+	+	+	-	+	-
rps16	+	+	+	-	+	-
rps18	+	+	+	+	+	+
rps19	+	+	+	+	+	+
rps2	+	+	+	+	+	+
rps3	+	+	+	+	+	+
rps4	+	+	+	+	+	+
rps7	+	+	+	+	+	+
rps8	+	+	+	+	+	+
rrn16S	+	+	+	+	+	+
rrn23S	+	+	+	+	+	+
rrn4.5S	+	+	+	+	+	+
rrn5S	+	+	+	+	+	+
trnA-UGC	+	+	+	-	+	+
trnC-GCA	+	+	+	+	+	Ψ
trnD-GUC	+	+	+	+	+	+
trnE-UUC	+	+	+	+	+	+
trnF-GAA	+	+	+	+	+	+
trnfM-CAU	+	+	+	+	+	-
trnG-GCC	+	+	-	+	+	-
trnG-UCC	+	+	+	-	+	-
trnH-GUG	+	+	+	-	+	+
trnI-CAU	-	-	+	+	-	+
trnI-GAU	+	+	+	-	+	+
trnK-UUU	+	+	+	-	+	-
trnL-CAA	+	+	Ψ	+	+	+
trnL-UAA	+	+	+	-	+	-
trnL-UAG	+	+	+	+	+	+
trnM-CAU	+	+	+	+	+	+
trnN-GUU	+	+	+	+	+	+
trnP-UGG	+	+	-	+	+	+
trnQ-UUG	+	+	+	+	+	+
trnR-ACG	+	+	+	+	+	+
trnR-UCU	+	+	+	+	+	Ψ
trnS-GCU	+	+	+	+	+	+
trnS-GGA	+	+	+	+	+	Ψ
trnS-UGA	+	+	+	+	+	+
trnT-GGU	+	+	+	+	+	-
trnT-UGU	+	+	+	+	+	-
trnV-GAC	+	+	+	+	+	-
trnV-UAC	+	-	-	-	-	-
trnW-CCA	+	+	+	+	+	+
trnY-GUA	+	+	+	+	+	+
ycf1	+	+	+	-	-	-
ycf2	+	+	+	+	+	+
ycf3	+	+	+	+	+	-
ycf4	+	+	+	+	+	-
ycf5	+	-	-	-	-	-
ycf9	+	-	-	-	-	-
ycf10	+	-	-	-	-	-
ycf15	+	+	Ψ	Ψ	-	-

Abbreviations: +, present; -, missing; Ψ, pseudogene

Table S2. Primer sequences at the verification of pseudogenes.

Gene	Forward/Reverse	Primer sequence (5′to 3′)
rpl16	F	AGGGCATATTGGGCATTGCG
	R	ATTCGCCCGCGAAAGTTTTT
ycf15	F	TCGACAGAGATGGCTTAGAACC
	R	TAACCCAGCAAGAAGCAGGG
rpl2	F	TAAGCCAGATGACGGAACGG
	R	TTTTAGCGGCTTGGCCTGTA

Table S3. Codon usage within the chloroplast genomes of *T. chinensis* and *T. sutchuenensis*.

Amino acid	Codon	No.		RSCU		Amino acid	Codon	No.		RSCU	
		①*	②*	①*	②*			①*	②*	①*	②*
Phe	TTT	676	685	1.36	1.38	Tyr	TAT	494	498	1.59	1.62
	TTC	318	308	0.64	0.62		TAC	127	117	0.41	0.38
Leu	TTA	596	603	2.09	2.12	Stop	TAA	48	38	1.41	1.84
	TTG	363	355	1.27	1.25		TAG	26	11	0.76	0.53
	CTT	341	330	1.20	1.16		TGA	28	13	0.82	0.63
	CTC	104	104	0.36	0.37	Trp	TGG	303	302	1.00	1.00
	CTA	207	205	0.73	0.72	Gln	CAA	470	466	1.49	1.49
	CTG	100	106	0.35	0.37		CAG	159	158	0.51	0.51
Ile	ATT	716	730	1.48	1.50	Asn	AAT	609	619	1.51	1.52
	ATC	260	269	0.54	0.55		AAC	198	197	0.49	0.48
	ATA	471	464	0.98	0.95	Lys	AAA	674	678	1.54	1.54
Met	ATG	386	375	1.00	1.00		AAG	201	200	0.46	0.46
Val	GTT	378	386	1.55	1.58	Asp	GAT	516	533	1.57	1.58
	GTC	103	101	0.42	0.41		GAC	140	142	0.43	0.42
	GTA	357	359	1.46	1.47	Glu	GAA	637	644	1.47	1.49
	GTG	137	134	0.56	0.55		GAG	227	223	0.53	0.51
Ser	TCT	365	353	1.73	1.67	Pro	CCT	235	229	1.36	1.35
	TCC	183	190	0.87	0.90		CCC	165	171	0.96	1.01
	TCA	235	237	1.11	1.12		CCA	193	181	1.12	1.07
	TCG	129	131	0.61	0.62		CCG	98	97	0.57	0.57
	AGT	270	278	1.28	1.32	Arg	CGT	216	234	1.28	1.36
	AGC	87	78	0.41	0.37		CGC	65	62	0.39	0.36
Cys	TGT	133	131	1.39	1.40		CGA	230	239	1.36	1.39
	TGC	58	56	0.61	0.60		CGG	84	81	0.50	0.47
Thr	ACT	375	376	1.69	1.68		AGA	294	297	1.74	1.73
	ACC	163	159	0.73	0.71		AGG	122	116	0.72	0.68
	ACA	258	266	1.16	1.19	His	CAT	340	338	1.51	1.51
	ACG	92	92	0.41	0.41		CAC	111	110	0.49	0.49
Ala	GCT	394	412	1.75	1.76	Gly	GGT	414	419	1.41	1.41
	GCC	153	163	0.68	0.70		GGC	116	114	0.39	0.38
	GCA	243	248	1.08	1.06		GGA	442	444	1.50	1.49
	GCG	112	111	0.50	0.48		GGG	204	212	0.69	0.71

* ①: *T. chinensis*; ②: *T. sutchuenensis*

Table S4. Simple sequence repeats (SSRs) in *T. chinensis* chloroplast genome.

SSR type	Repeat motif	Size (bp)	Start	End	Region
p1	(A)10	10	7215	7224	LSC
p1	(A)10	10	51768	51777	LSC
p1	(A)10	10	93998	94007	SSC
p1	(A)11	11	3035	3045	LSC
p1	(A)11	11	36782	36792	LSC
p1	(A)12	12	76661	76672	IRa
p1	(A)13	13	57250	57262	LSC
p1	(A)14	14	98749	98762	SSC
p1	(A)8	8	8442	8449	LSC
p1	(A)8	8	17810	17817	LSC
p1	(A)8	8	25843	25850	LSC
p1	(A)8	8	29431	29438	LSC
p1	(A)8	8	39660	39667	LSC
p1	(A)8	8	50350	50357	LSC
p1	(A)8	8	57551	57558	LSC
p1	(A)8	8	90683	90690	IRa
p1	(A)8	8	94784	94791	SSC
p1	(A)8	8	95620	95627	SSC
p1	(A)9	9	9125	9133	LSC
p1	(A)9	9	17187	17195	LSC
p1	(A)9	9	24284	24292	LSC
p1	(A)9	9	35315	35323	LSC
p1	(A)9	9	38374	38382	LSC
p1	(A)9	9	46981	46989	LSC
p1	(A)9	9	56658	56666	LSC
p1	(A)9	9	58046	58054	LSC
p1	(A)9	9	59596	59604	LSC
p1	(A)9	9	94189	94197	SSC
p1	(A)9	9	105569	105577	IRb
p1	(C)10	10	55503	55512	LSC
p1	(C)8	8	19968	19975	LSC
p1	(C)8	8	43314	43321	LSC
p1	(C)8	8	90129	90136	IRa
p1	(G)8	8	101581	101588	IRb
p1	(T)10	10	1602	1611	LSC
p1	(T)10	10	4004	4013	LSC
p1	(T)10	10	4778	4787	LSC
p1	(T)10	10	13342	13351	LSC
p1	(T)10	10	28650	28659	LSC
p1	(T)10	10	37692	37701	LSC
p1	(T)10	10	57048	57057	LSC
p1	(T)10	10	61379	61388	LSC

p1	(T)10	10	70346	70355	LSC
p1	(T)10	10	98108	98117	SSC
p1	(T)11	11	62990	63000	LSC
p1	(T)11	11	94971	94981	SSC
p1	(T)12	12	115045	115056	IRb
p1	(T)14	14	8840	8853	LSC
p1	(T)8	8	2037	2044	LSC
p1	(T)8	8	9486	9493	LSC
p1	(T)8	8	11434	11441	LSC
p1	(T)8	8	48465	48472	LSC
p1	(T)8	8	51553	51560	LSC
p1	(T)8	8	55182	55189	LSC
p1	(T)8	8	59449	59456	LSC
p1	(T)8	8	65790	65797	LSC
p1	(T)8	8	68047	68054	LSC
p1	(T)8	8	69243	69250	LSC
p1	(T)8	8	94631	94638	SSC
p1	(T)8	8	95839	95846	SSC
p1	(T)8	8	101027	101034	IRb
p1	(T)9	9	68	76	LSC
p1	(T)9	9	3526	3534	LSC
p1	(T)9	9	10362	10370	LSC
p1	(T)9	9	13202	13210	LSC
p1	(T)9	9	20333	20341	LSC
p1	(T)9	9	35178	35186	LSC
p1	(T)9	9	35513	35521	LSC
p1	(T)9	9	36422	36430	LSC
p1	(T)9	9	37560	37568	LSC
p1	(T)9	9	55855	55863	LSC
p1	(T)9	9	57829	57837	LSC
p1	(T)9	9	61126	61134	LSC
p1	(T)9	9	69064	69072	LSC
p1	(T)9	9	86140	86148	IRa
p2	(AG)4	8	102712	102719	IRb
p2	(AT)4	8	25647	25654	LSC
p2	(AT)4	8	40716	40723	LSC
p2	(AT)4	8	43446	43453	LSC
p2	(AT)4	8	49990	49997	LSC
p2	(AT)4	8	66094	66101	LSC
p2	(AT)5	10	38043	38052	LSC
p2	(AT)5	10	52685	52694	LSC
p2	(AT)5	10	69603	69612	LSC
p2	(AT)5	10	111658	111667	IRb
p2	(CA)4	8	108032	108039	IRb

p2	(CT)4	8	18379	18386	LSC
p2	(CT)4	8	49096	49103	LSC
p2	(CT)4	8	88998	89005	IRa
p2	(GA)4	8	44277	44284	LSC
p2	(GA)4	8	72281	72288	IRa
p2	(GA)4	8	105094	105101	IRb
p2	(TA)4	8	1396	1403	LSC
p2	(TA)4	8	14725	14732	LSC
p2	(TA)4	8	39233	39240	LSC
p2	(TA)4	8	39829	39836	LSC
p2	(TA)4	8	72506	72513	IRa
p2	(TA)4	8	91116	91123	IRa
p2	(TA)4	8	100594	100601	IRb
p2	(TA)4	8	119204	119211	IRb
p2	(TA)5	10	80049	80058	IRa
p2	(TC)4	8	67559	67566	LSC
p2	(TC)4	8	86616	86623	IRa
p2	(TC)4	8	119429	119436	IRb
p2	(TG)4	8	45510	45517	LSC
p2	(TG)4	8	83678	83685	IRa
p3	(AAG)4	12	83225	83236	IRa
p3	(CTT)4	12	108481	108492	IRb
p4	(AACA)3	12	68524	68535	LSC
p4	(GTCT)3	12	6664	6675	LSC

Table S5. Simple sequence repeats (SSRs) in *T. sutchuenensis* chloroplast genome.

SSR type	Repeat motif	Size (bp)	Start	End	Region
p1	(A)10	10	29043	29052	LSC
p1	(A)10	10	50942	50951	LSC
p1	(A)10	10	51727	51736	LSC
p1	(A)11	11	39352	39362	LSC
p1	(A)12	12	76919	76930	IRa
p1	(A)13	13	24237	24249	LSC
p1	(A)13	13	57202	57214	LSC
p1	(A)14	14	99497	99510	SSC
p1	(A)8	8	3070	3077	LSC
p1	(A)8	8	3637	3644	LSC
p1	(A)8	8	7103	7110	LSC
p1	(A)8	8	8334	8341	LSC
p1	(A)8	8	17728	17735	LSC
p1	(A)8	8	36823	36830	LSC
p1	(A)8	8	39773	39780	LSC
p1	(A)8	8	50384	50391	LSC
p1	(A)8	8	80902	80909	IRa
p1	(A)8	8	93344	93351	IRa
p1	(A)8	8	94729	94736	SSC
p1	(A)8	8	95556	95563	SSC
p1	(A)8	8	96422	96429	SSC
p1	(A)8	8	106441	106448	IRb
p1	(A)9	9	4097	4105	LSC
p1	(A)9	9	17102	17110	LSC
p1	(A)9	9	25700	25708	LSC
p1	(A)9	9	39537	39545	LSC
p1	(A)9	9	47020	47028	LSC
p1	(A)9	9	58292	58300	LSC
p1	(A)9	9	59499	59507	LSC
p1	(C)8	8	19881	19888	LSC
p1	(G)8	8	45019	45026	LSC
p1	(T)10	10	3325	3334	LSC
p1	(T)10	10	9199	9208	LSC
p1	(T)10	10	9391	9400	LSC
p1	(T)10	10	13299	13308	LSC
p1	(T)10	10	61700	61709	LSC
p1	(T)11	11	47327	47337	LSC
p1	(T)11	11	95743	95753	SSC
p1	(T)12	12	56427	56438	LSC
p1	(T)12	12	116263	116274	IRb
p1	(T)8	8	8761	8768	LSC

p1	(T)8	8	11387	11394	LSC
p1	(T)8	8	29750	29757	LSC
p1	(T)8	8	35175	35182	LSC
p1	(T)8	8	38367	38374	LSC
p1	(T)8	8	48507	48514	LSC
p1	(T)8	8	55109	55116	LSC
p1	(T)8	8	57002	57009	LSC
p1	(T)8	8	59352	59359	LSC
p1	(T)8	8	63370	63377	LSC
p1	(T)8	8	69498	69505	LSC
p1	(T)8	8	86745	86752	IRa
p1	(T)8	8	99842	99849	IRb
p1	(T)8	8	112284	112291	IRb
p1	(T)9	9	1647	1655	LSC
p1	(T)9	9	2072	2080	LSC
p1	(T)9	9	7566	7574	LSC
p1	(T)9	9	13155	13163	LSC
p1	(T)9	9	20246	20254	LSC
p1	(T)9	9	21922	21930	LSC
p1	(T)9	9	29172	29180	LSC
p1	(T)9	9	35533	35541	LSC
p1	(T)9	9	48906	48914	LSC
p1	(T)9	9	57753	57761	LSC
p1	(T)9	9	61035	61043	LSC
p1	(T)9	9	69319	69327	LSC
p1	(T)9	9	70457	70465	LSC
p1	(T)9	9	96631	96639	SSC
p2	(AG)4	8	103534	103541	IRb
p2	(AT)4	8	50024	50031	LSC
p2	(AT)7	14	37236	37249	LSC
p2	(CA)4	8	108848	108855	IRb
p2	(CT)4	8	18292	18299	LSC
p2	(CT)4	8	23153	23160	LSC
p2	(CT)4	8	89652	89659	IRa
p2	(GA)4	8	44284	44291	LSC
p2	(GA)4	8	72563	72570	IRa
p2	(GA)4	8	105953	105960	IRb
p2	(TA)4	8	1395	1402	LSC
p2	(TA)4	8	4461	4468	LSC
p2	(TA)4	8	14672	14679	LSC
p2	(TA)4	8	25864	25871	LSC
p2	(TA)4	8	36204	36211	LSC
p2	(TA)4	8	39156	39163	LSC
p2	(TA)4	8	70992	70999	IRa

p2	(TA)4	8	72788	72795	IRa
p2	(TA)4	8	91775	91782	IRa
p2	(TA)4	8	101411	101418	IRb
p2	(TA)4	8	120398	120405	IRb
p2	(TA)4	8	122194	122201	IRb
p2	(TA)5	10	53052	53061	LSC
p2	(TA)7	14	38006	38019	LSC
p2	(TC)4	8	87233	87240	IRa
p2	(TC)4	8	120623	120630	IRb
p2	(TG)4	8	45544	45551	LSC
p2	(TG)4	8	84338	84345	IRa
p3	(AAG)4	12	83889	83900	IRa
p3	(CTT)4	12	109293	109304	IRb
p4	(GTCT)3	12	6552	6563	LSC
p4	(TAAG)3	12	51172	51183	LSC

Table S6. Primer sequences at the discrepancies in assembly results obtained using Illumina and PacBio.

No.	Sites (bp)	Forward/Reverse	Primer sequence (5′to 3′)
1	4615	F	GTTTGGCAAGCCGCTCTAAG
		R	ACCTATCCGGGCTGTATTGC
2	17326	F	GATCGGTCTTAGTTCGGGGG
		R	ATCGCGTAGAGGCTTGAAGG
3	17952	F	CCCTTCGAATCATGAATCCGC
		R	TTGGCTCGGTTTCTCCTCAA
4	27768	F	CTTGGTGGTCGGGAAATGC
		R	TTGAGCGGATCCCACGTT
5	32700	F	CCACAGCCGATTGACCAAAA
		R	CGTGTGATGGACCTGGAAGA
6	41040	F	TGGCACAGGATGATCAATTTGA
		R	ATCACGCCCCTATTGCTACA
7	45020	F	GCAGGTACATGCGAAGAAATG
		R	GATCCAAGGCCCGAAACTAC
8	51046	F	GACCGGAACTCCTTGTTTCG
		R	GTCTCTAGCAGGACCACTTGAT
9	55514	F	CTCCTCGAAAGCTTGCTGC
		R	GGTGTATGTGCGACTCGTTC
10	58056	F	CTCCGGGTAAAGATCCGACC
		R	TCAAAGGACCCGCCTATTCG
11	87056	F	TCGGGGAGGATAAATCGCTC
		R	TGTTTCAGTTCGCCAGGTTG
12	90138	F	CAAAGGCAGAAGGGAGCTTG
		R	CTCTACGCCTAGGACACCAG
13	101590	F	CTCTACGCCTAGGACACCAG
		R	CAAAGGCAGAAGGGAGCTTG
14	104671	F	TGTTTCAGTTCGCCAGGTTG
		R	TCGGGGAGGATAAATCGCTC

Table S7. Primer sequences at the boundaries between single copy and IR regions.

Species	Regions	Forward/Reverse	Primer sequence (5′ to 3′)
Taxillus chinensis	LSC-IRa	F	GGTAGATGCTCGTGACCAAG
		R	ACATAAGCCAGATGACGGAAC
	IRa-SSC	F	GTTTCGATGCAACAACAAGATG
		R	GGAGAAACCCCATGAAAGGA
	SSC-IRb	F	CACCAAAAGATTGGTTCAATAAAGG
		R	TGTTTCGATGCAACAACAAGAT
	IRb-LSC	F	TAAGCCAGATGACGGAACGG
		R	TTTTAGCGGCTTGGCCTGTA
Taxillus sutchuenensis	LSC-IRa	F	AATGGTAGATGCTCGTGACC
		R	CATAAGCCAGATGACGGAACG
	IRa-SSC	F	GTTTCGATGCAACAACAAGATG
		R	GGAGAAACCCCATGAAAGGA
	SSC-IRb	F	AGGTTGGTTCAAGAAAGGATCA
		R	GTTTCGATGCAACAACAAGATG
	IRb-LSC	F	GGAACGGGAAGACCTAGGATG
		R	CGGCTTGGCCTGTAGTAGG
